# Community pharmacy teams’ experiences of general practice-based pharmacists: an exploratory qualitative study

**DOI:** 10.1186/s12913-020-05245-y

**Published:** 2020-05-18

**Authors:** Georgios Dimitrios Karampatakis, Nilesh Patel, Graham Stretch, Kath Ryan

**Affiliations:** 1 School of Pharmacy, University of Reading, Whiteknights Campus, PO Box 226, Reading, RG6 6AP UK; 2 Ealing GP Federation, 179C Bilton Road, Perivale, Greenford, Middlesex UB6 7HQ UK

**Keywords:** Community pharmacy, England, General practice, Experiences, Qualitative study

## Abstract

**Background:**

In England, since 2015, there has been a formal drive to integrate pharmacists into general practice as a new healthcare service. Research efforts have offered insights into how general practice-based professionals and patients view the service, however, they took no account of community pharmacy teams’ opinions. There have been anecdotal statements about opposition from community pharmacies to the service, due to fears of losing business. The aim of the current study was to identify the experiences and perceptions of community pharmacy teams regarding pharmacists’ presence in general practice.

**Methods:**

The National Health Service Choices website was used to identify community pharmacies within a radius of two miles from eight West London general practices. The search resulted in 104 community pharmacies which were all contacted via telephone. Pharmacy staff who verbally expressed their interest to participate were then provided with the study’s documents. Qualitative, face-to-face, semi-structured interviews were conducted inside the pharmacy from which each participant was recruited. Interviews lasted 30 to 45 min and were audio-recorded. Audio-recordings were transcribed verbatim and transcripts analysed thematically.

**Results:**

Forty-eight community pharmacy staff participated. Four themes were discerned: awareness (“I knew that [pharmacists] have already been implemented [in general practice] but I haven’t really followed it … where does the pharmacist role come?”); interactions (“I’m just so pleased that there’s a pharmacist professional in the general practice … because we speak the same language!”); patient care (“if I was a patient knowing that there is a general practitioner and a pharmacist [in general practice], I would … think ‘nothing can go wrong at the moment’”); and funding challenges (“if general practices take on the extra responsibility of stop smoking or flu vaccination campaigns … financially, this would affect this pharmacy”).

**Conclusions:**

The current study revealed the perceived impact of general practice-based pharmacists on community pharmacies would be improved communication between pharmacies and practices. Findings will inform policy so that any future framing of pharmacists’ presence in general practice considers the needs of community pharmacies.

## Background

England is experiencing pressures in the delivery of primary healthcare due to an ageing population with multiple co-morbidities and significant difficulties in recruiting and retaining general practitioners (GPs) [1, 2]. To address these pressures and make effective use of the workforce (including a projected excess of recently qualified pharmacists by 2040 [3]), a national, two-phased scheme for integrating pharmacists into general practice was launched in 2015 by the National Health Service (NHS) England [4, 5]. The scheme introduced over 1000 pharmacists into general practice [6]. In early 2019, the NHS Long Term Plan [7] encouraged general practices to work together in Primary Care Networks (PCNs). PCNs are organisations covering 30–60,000 patients with the remit to connect community-based healthcare services with each other as well as with the hospital, social and voluntary sectors. As part of a new contract between general practices and NHS England, PCNs will be funded £1.8 billion to hire an additional 20,000 primary care staff within the next 5 years including large numbers of general practice-based pharmacists [8]. General practice-based pharmacists in England carry out a wide spectrum of activities, the majority of which focus on providing expertise around medication use [9–12]. Characteristic examples of activities are face-to-face patient clinics for managing long-term conditions and/or acute care and/or medication reviews, including elements of physical assessments; drug monitoring including dealing with high-risk drugs and ordering laboratory or other clinical tests; lifestyle advice, for example, weight management, diet and smoking cessation; telephone consultations with patients for triage and common ailments such as management of pain; medicines reconciliations; prescribing, if qualified to do so; managing the repeat prescription service; and advising general practice-based staff on medication-related queries.

Internationally, several countries have formally attempted to integrate pharmacists into general practice. For example, several programmes have been introduced in different parts of Australia, all of which have been small-scale [13–16]. In Canada, large-scale programmes of implementing pharmacists’ services into general practice have taken place in Ontario and Quebec [17, 18] whereas smaller efforts have been also made in Newfoundland and Labrador [19]. In the USA, efforts of introducing general practice-based pharmacists have involved a small number of pharmacists/practices and have been restricted to a very certain geographical location [20–24]. However, there are a few instances of wider programmes such as the ‘Collaboration Among Pharmacists and Physicians to Improve Outcomes Now’ which ran across 13 states [25]. In New Zealand, governmental programmes implemented 31 general practice-based pharmacists nationwide by 2017 [26]. In Netherlands, a relatively recent initiative introduced ten general practice-based pharmacists [27].

Although some pharmacists in England have occasionally worked in general practice in the past [28, 29], this is the first time that the role has been formally implemented and tested at a national level [30]. As a result, very little is known about the impact of pharmacists in general practice. A preliminary evaluation of the first phase of the English scheme reported benefits for GPs (including increased capacity and a more focused workload), patients (including longer appointments with the same person) and pharmacists (including increased role satisfaction) [31]. The evaluation, however, did not account for the perceptions of community pharmacy staff. Staff in community pharmacies mainly consists of pharmacists, including pre-registration pharmacists, and pharmacy technicians.

Community pharmacies are independent contractors of NHS England and are integral to the care of patients. There are currently about 11,600 community pharmacies across England [32]. Many of these pharmacies operate long hours, hence providing patient care at times when other healthcare services are unavailable and/or serve small, deprived communities where access to healthcare is hard.

As part of their contract, community pharmacies provide three tiers of services, namely ‘essential’, ‘advanced’ and ‘locally commissioned’ [33–35]. All these services are carried out by pharmacists. The ‘essential’ services are required from all community pharmacies whereas the ‘advanced’ and ‘locally commissioned’ services are provided on an optional basis. The ‘essential’ services include dispensing duties, liaising with other healthcare settings when needed (in particular general practices), disposal of medical waste and referral of patients to the appropriate healthcare professional. ‘Advanced’ services refer to tasks such as the adherence-focused medicine use reviews (MURs), to be phased out by the end of 2020; the new medicine service (NMS); flu vaccinations; and providing emergency medication supplies to patients. Community pharmacies receive payment for offering ‘advanced’ services and as such employers expect pharmacists to meet certain targets for their provision. ‘Locally commissioned’ services include minor ailment, smoking cessation, lifestyle advice and vascular risk assessment services. Pharmacy technicians, under the supervision of a pharmacist, provide various services in community pharmacies, for example, processing prescriptions, preparing and dispensing medications, ordering items and managing stocks, liaising with staff in other healthcare settings and advising patients on their medications as well as on minor ailments and smoking cessation [36, 37].

There are existing interactions between community pharmacies and general practices, mainly around prescriptions [38, 39]. However, the increase in number of pharmacists in general practice and the roles they can undertake (for example, medication reviews) has anecdotally caused some opposition and resistance from community pharmacy teams to the integration of pharmacists into general practice. These rumours are based on fears of losing business due to role clashes, for example, general practice-based pharmacists’ medication reviews replacing MURs and the NMS [40, 41]. A few, mostly international studies examining collaboration between community pharmacies and general practice focused on the views of general practice-based pharmacists and other staff, rather than on the opinions of community pharmacy teams [42–46]. Therefore, there is a gap in the literature examining the impact of general practice-based pharmacists on community pharmacies.

The aim of the current study was to explore community pharmacy teams’ experiences and perceptions of the presence of pharmacists in general practice in England.

## Methods

### Study design

To pursue an in-depth exploration of experiences, a qualitative study design was selected. Individual interviews, rather than focus groups, were selected as participants might not have felt comfortable to discuss their honest views in front of other co-participants, especially if there were areas of disappointment/frustration. All interviews were conducted between October and December 2017.

### Setting

Participants were recruited from community pharmacies within the geographical area of one West London GP Federation (a cohort of practices working together as a collective entity) which constituted a ‘pharmacists in general practice’ site. At the time of data collection, eight Federation practices participated in the scheme, employing seven pharmacists and serving approximately 72,000 patients. This Federation was targeted as a recruitment point due to working connections with the research team’s organisation.

### Participants and recruitment

Potential participants included community pharmacists (either regular or locum), pre-registration pharmacists and pharmacy technicians. Pharmacy technicians were included because they are an important part of community pharmacy teams with expanding roles and responsibilities that support pharmacists in their roles [47, 48]. The NHS Choices website was searched to identify all community pharmacies within a two-mile radius of the postcodes of the eight practices. This identified 104 pharmacies (61 independent, 24 small chain, 13 large chain and six supermarket pharmacies). Names, addresses and phone numbers for the pharmacies were retrieved. Ten interviewers (final year pharmacy students trained to undertake interviews) were paired and each pair was randomly assigned 20 or 21 pharmacies. Each pharmacy was contacted by telephone and the responsible pharmacist introduced to the study and asked if they, or a member of their staff, were willing to participate. People who expressed their willingness to participate were provided, by e-mail or post, with information about the study including details on the research team and interviewers. After a week, invitees were again contacted by telephone to schedule an interview.

### Data collection

Participation in the interviews was voluntary. Regular debriefings between the research team (GDK, KR, NP and GS) and the interviewers were held throughout the data collection period. GDK is a doctoral research student, KR is a Professor of Social Pharmacy, NP is an Associate Professor in Pharmacy and GS is a lead general practice-based pharmacist. All interviews were face-to-face, semi-structured, audio-recorded and conducted within the pharmacies in a quiet place of mutual convenience. An interview schedule, composed of open-ended questions and prompts, was used (see Table 1). Each interview terminated when the participant did not have anything else to share. Interviews lasted 30 to 45 min.

**Table 1:** Interview schedule

Thank you for taking the time to contribute to our research project.	
1. What are your roles and responsibilities within this pharmacy?	
• Your role, age, years of service	
• Skills and training	
• Day-to-day working life	
2. Please tell us about your perceptions of pharmacists working in general practice.	
• Positives/negatives	
• Perceived impact on your own roles, responsibilities	
• Actual experiences or hearsay (where from?)	
3. Please tell us about your experiences, if any, of the pharmacists in general practice scheme	
• Relationships – with GPs, pharmacy team members	
• Positive/negatives - examples	
• Impact on own roles, responsibilities	
4. Overall view of the GP-pharmacist partnership on your work/services provided	
• Feelings	
• Thoughts about how your work has changed	
• What changes would you like to see made to the scheme?	

### Data analysis

Audio-recordings were transcribed verbatim by the interviewers. Transcripts were inductively coded [49] using NVivo 11 and analysed thematically by GDK as described by Braun and Clark (familiarisation with data; coding; searching for themes; reviewing themes; defining and naming themes; and developing the report) [50]. Coding was verified by the rest of the research team before developing categories. Categories were re-assessed and collapsed into potential themes with associated sub-themes. Themes were then refined and named collectively by the research team. Participants’ feedback on transcripts or findings was not sought.

## Results

### Demographics

Table 2 provides an overview of the participants’ demographics. Forty-eight community pharmacy staff participated in the study. Of these, most were pharmacists with a wide range of time spent in community pharmacy. The majority of participants worked for independent pharmacies.

**Table 2:** Demographics of participants

	Total number interviewed	Years of experience in community pharmacy
**Pharmacists**	32	4 months to 44 years
**Pre-registration pharmacists**	8	2 to 5 months
**Pharmacy technicians**	8	1 to 30 years

### Themes

**Fig. 1:**
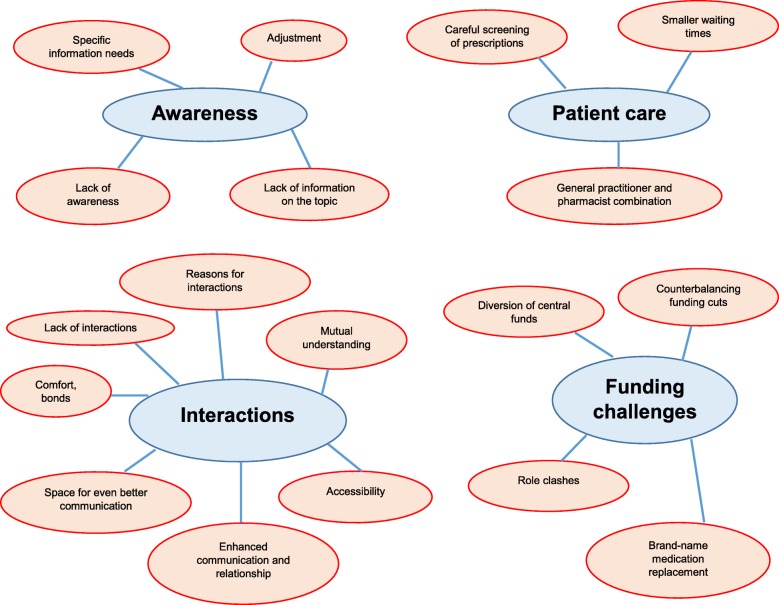
Themes and subthemes of community pharmacy teams’ experiences of pharmacists in general practice

Four overarching themes were discerned in the data: awareness; interactions; patient care; and funding challenges. Figure 1 illustrates the four themes with all associated sub-themes.

### Awareness

Participants’ awareness of pharmacists in general practice varied. Some were completely ignorant of their presence or thought that a general practice-based pharmacist meant a pharmacy situated within the practice.

*To be honest, I don’t know anything about the scheme [of integrating pharmacists into general practice] so I’m not able to speak about it.* (Pharmacist 25)

The majority of participants, though, were aware of pharmacists’ presence in general practice but uncertain about details such as employment models, roles and responsibilities.

*I knew that [pharmacists] have already been implemented [in general practice] but I haven’t really followed it … where does the pharmacist role come? For example, I don’t know if they see the patient [together] with the GP or if they have their own room or like clinic or whatever where they can see patients.* (Pharmacist 28)

Participants attributed their limited awareness of the topic to a lack of formal publicity about the scheme. They reported, for example, that general practices had not informed community pharmacies about the integration of a pharmacist or the specific roles this pharmacist would undertake.

*General practices never told community pharmacy teams that there’s going to be a pharmacist integrated into them. It’s just when we see a prescription that has a name and pharmacist prescriber [that we understand there is a pharmacist in a practice]. And then, there was one incident where the pharmacist [in general practice] actually phoned [us] for some information and that was the first time we knew that the practice had a pharmacist.* (Pharmacist 10)

Participants also reported receiving no information on the scheme from NHS England or pharmacy professional bodies, including upcoming rounds of recruitment, number of posts, job security matters or the longevity of the role. They said they were opportunistically updated on the topic via self-research, such as reading online documents including job applications, or through friends or family working in general practice.

*There is not a lot of information about pharmacists in general practice out there. No General Pharmaceutical Council (GPhC) [the responsible body for regulating the pharmacy profession in England, Scotland and Wales] e-mails, nothing whatsoever. I think that if GPhC wants this [pharmacists’ integration into general practice] to go through, they have to advertise it more. We have so little update from them!* (Pharmacy technician 8)

Participants highlighted the importance of being made aware of the presence of a pharmacist in a general practice so that they had some time to adjust and avoid awkwardness.

*It’s like ‘are we being told off’? But ultimately you’d just have to think that if it’s to do with patient care and streamlining everything and making things quicker, as long as we’re told that there’s a pharmacist now employed [in a general practice] to do this and this.* (Pharmacist 2)

Community pharmacy teams had specific information needs including the time scales for recruiting general practice-based pharmacists; the practices involved; the precise targets that a practice aims to achieve with the integration of a pharmacist; the benefits for the surrounding community pharmacies; and the times during which pharmacists are available in a practice, especially for pharmacists covering multiple practices.

### Interactions

There were a few participants without any sort of interaction with a general practice-based pharmacist.

*I know that some of our prescriptions come from a pharmacist prescriber in general practice. But I have never personally spoken to that person.* (Pharmacist 31)

Queries and/or problems on prescriptions issued by general practices were the main reasons for interactions, usually over the telephone, between community pharmacy teams and general practice-based pharmacists. These queries included missing items on a repeat prescription, need for dose amendments and need for an alternative prescription if a product was out of stock.

*[Community pharmacies] do dosette boxes [for storing scheduled doses for a patient’s medications to aid adherence]. When there has to be changes in the dosette boxes, we speak to the general practice-based pharmacist. So, that’s the main interaction we have: any prescription they [general practice-based pharmacists] issue, if there’s a query in that prescription like a dose amendment then we speak to them.* (Pharmacist 1)

Participants emphasised their satisfaction about being able to speak to another pharmacist on the telephone when engaging with general practices. Community pharmacists, including pre-registration pharmacists, felt more comfortable and at ease speaking to another pharmacist than speaking to a GP. They attributed this feeling to intra-professional bonds that make interactions with somebody at the same level and with similar training easier.

*When I call [doctors] there is a bit of clash and I just feel if I speak to a pharmacist [in general practice] it’s so much easier. There is always that awkwardness between doctors and pharmacist. For example, I was once on the phone with a doctor and he said ‘I have no time for this’. I would not say that to somebody.* (Pharmacist 21)

Fellow pharmacists were thought to fully understand the business side of community pharmacy and its implications, as opposed to receptionists or GPs who have historically dealt with queries from community pharmacy teams.

*It’s great that I can pick up the phone and speak to a pharmacy member of [general practice] staff who understands what I mean by dosette or why I need a prescription. For example, you have ordered ten items for a repeat and [general practice] issue nine. Receptionists will say ‘I will do it tomorrow or the day after, it’s not urgent’. They don’t realize it’s popped in the dosette and it needs to go at the same time. Receptionists sometimes don’t get these basic things because to them that’s foreign stuff. But in the world of pharmacy that’s just minor.* (Pharmacist 20)

*I’m just so pleased that there’s a pharmacist professional in the general practice. I’m so happy about it because we speak the same language! GPs are so remotely unaware of many things [in community pharmacy].* (Pharmacist 3)

Participants also highlighted that it is easier to contact a pharmacist in general practice than a GP. Quick access resulted in resolution of queries and timely issue of prescriptions. The presence, therefore, of pharmacists in general practice was believed to reduce workload stress on community pharmacy teams by streamlining the dispensing process and avoiding multiple phone calls to practices.

*I feel the pharmacist [in general practice] is more accessible than a doctor, because obviously doctors are very busy. For example, if I want to query a patient’s dose [on a prescription issued by the practice] it’s very easy to get through to a pharmacist. Because by the time we contact and get to a doctor and the receptionist is like ‘The doctor is busy, I don’t know when they can call you, it can be today, it can be tomorrow’. But with the pharmacist it is ‘Okay, let’s do this right now’. I get a quick reply, so it’s very good.* (Pharmacy technician 3)

Conversely, a few participants reported occasional difficulties in accessing the pharmacist in general practice, citing the absence of a direct line and variable availability. Moreover, said one participant, high workload pressures led general practice-based pharmacists to refuse to deal with more than one query at a time.

Participants thought pharmacists’ presence in general practice has resulted in enhanced communication and better relationships between community pharmacies and general practices. For instance, practices with an integrated pharmacist were thought to more readily consider reports from community pharmacies, for example, investigating identified cases of hypnotics’ overuse. In addition, they thought that GPs, who employed a pharmacist, were more likely to ask community pharmacists for guidance on prescribing.

*There can be a real communication gap between surgeries and [community] pharmacies. This gap closes down having [a pharmacist] be part [of] that setting. And then the general practices that have a clinical pharmacist on site, they’re always very welcoming and always more willing to help and communicate with a pharmacist that’s calling out from the community because they appreciate [the pharmacist’s] role.* (Pharmacist 5)

One participant, however, mentioned that practices covered by the same pharmacist refused to handle prescription requests at times when pharmacists were not physically present in the practice. Participants also noted that there is still room for improved communication between community pharmacies and general practices. For instance, participants emphasised the importance of establishing face-to-face interactions and familiarity with general practice staff as a springboard for closer working relationships and mutual support.

*I think the working relationships will improve a lot more if community pharmacy teams could actually face-to-face meet these people [general practice-based staff], with whom we speak on the phone. For example, they could tell us what their problems are and we can see if we can make any changes to make their lives easier. And we can tell them what our problems are and they can help sort them out. At the end of the day you have one neutral person, the patient.* (Pharmacist 19)

### Patient care

Participants thought that the integration of pharmacists into general practice has led to improved satisfaction and better quality of care for patients. For example, faster processing of prescriptions meant shorter waiting times and enhanced safety, especially patients’ ability to receive antibiotics and emergency medicines quickly.

Participants were confident that general practice-based pharmacists clinically screen all prescriptions before they are signed and forwarded to community pharmacies. They were not confident that screening happened when receptionists and GPs processed prescriptions. Careful screening was believed to identify prescription problems, such as incorrect doses, obsolete items and unsynchronised repeat medication. Screening could also identify unsatisfactory adherence, potential interactions and adverse effects that otherwise might have been missed.

*General practice-based pharmacists pick up more than what the GP would, for example … if we request a prescription [from the practice], the receptionist puts the prescriptions down and then the doctor just rush-signs them whereas a pharmacist on site takes more time looking at things and realising that patients are actually overusing or underusing [medicines].* (Pharmacy technician 2)

GPs’ skills in diagnostics combined with pharmacists’ expertise in medications was perceived to be an ideal approach to patient care.

*If I was a patient knowing that there is a GP and a pharmacist [in general practice], I would kind of automatically think ‘Nothing can go wrong at the moment, two professions combining together that must be the correct thing for me’.* (Pre-registration pharmacist 6)

### Funding challenges

There were concerns that pharmacists’ integration into general practice was associated with cuts in the funding of community pharmacies. Participants thought, without corroborating evidence, that there had been a diversion of central funds from community pharmacies to general practices to support the new role.

*I know that there definitely have been cuts because I know some pharmacies have been closed down. So that is definitely true, but, where policymakers have actually put the money obviously no one knows. But because this is a new role, it makes sense that they’re just kind of pushing funding all in there.* (Pharmacist 12)

There were also fears that certain services traditionally carried out in community pharmacies could potentially be provided by general practice-based pharmacists, thereby negatively affecting the profitability of community pharmacies. Examples included MURs and other patient-facing services.

*If, for example, general practices take on the extra responsibility of stop smoking or flu vaccination campaigns, then it is possible that some of the patients who would have normally come to my pharmacy will go to the practice for it. General practices have the capacity now to do these additional services because of an extra pharmacist. Financially, this would affect this pharmacy. If a general practice has done a smoking cessation service, they would get the registration fee, they would get the quit [compensation] and yet I would be providing the medication. So, all I get would be the cost of medication and nothing else.* (Pharmacist 26)

Finally, the widespread use of generic medications because of the presence of general practice-based pharmacists meant reduced income for community pharmacies due to lower reimbursements.

*I have personally seen a drop in our remuneration and it pinches you. This is because general practice-based pharmacists try and put more generics everywhere, if they can, because a lot of the drugs initially were all branded and now it’s basically as many generics. So, it’s less income [for us] because obviously the brands are a lot more expensive and increase our turnover overall.* (Pharmacist 24)

To counterbalance funding reductions, participants claimed that they would have to increase over-the-counter medication sales, and pursue additional qualifications (for example, clinical diplomas) to increase the range of services they could offer.

## Discussion

Findings indicate a lack of awareness amongst community pharmacy teams of various aspects of general practice-based pharmacy services. Conversely, improved communication has resulted in more timely and safer patient care. The financial viability of community pharmacy, however, is still a concern. Despite these few concerns, the majority of participating community pharmacy staff did not oppose to the scheme and were supportive of pharmacists’ integration into general practice.

The findings can be interpreted using the ‘structuration model of collaboration’ developed by D’Amour and colleagues [51]. This theoretical framework analyses intra- and inter-professional collaboration in healthcare. It consists of four dimensions, two of which (internalisation; shared goals and vision) examine relationships between professionals and two (governance; formalisation) that examine organisational aspects influencing collaboration.

If we take the dimensions that examine relationships between professionals, in the current study these would refer to the relationships between community pharmacy teams (participants) and general practice-based pharmacists. These two cohorts of people will be our collaborating parts of interest, which in the ‘structuration model of collaboration’ are described as the ‘actors’ in collaboration. To begin with internalisation (composed of trust and acquaintanceship), in our study, was obtained by intra-professional bonds between ‘actors’ and confidence that general practice-based pharmacists functioned as a safety net on prescriptions. Participants’ sense of ‘having one of them in general practice’ was the element that bridged gaps between organisations (practices and pharmacies). Barriers to internalisation were participants’ insufficient knowledge of roles and absence of face-to-face acquaintanceship between ‘actors’. Participants conceptualised the dimension of shared goals and vision by quoting the benefit of the patient as the reason for both ‘actors’ to tighten relationships. It is unclear, however, whether or not ‘actors’ had the chance to explicitly communicate this goal since most mutual interactions were brief, non-face-to-face discussions on prescription matters. Potential failure of both ‘actors’ to focus on the interests of the patient could activate allegiances to personal interests (such as participants’ keenness to defend their existing funding status) that could preclude mutual understanding.

Regarding organisational dimensions, formalisation (entailing how the clinical care is structured) was indicated in the way general practice-based pharmacists were easily accessible, often via direct telephone lines, which facilitated communication between ‘actors’. The dimension of governance (leadership functions promoting collaboration) was illustrated in participants’ eagerness to take the lead and secure relationships. Both organisational dimensions, however, were hindered by scarce official initiatives. For example, practices did not divulge pharmacists’ integration which surprised participants who had limited time to adjust (including getting used to ways of offsetting income reductions). Some participants even ignored the presence of the other ‘actor’ making collaboration impossible. Additionally, lack of information from professional bodies hindered participants’ understanding of the full benefits of the scheme and generated suspicions about role clashes.

Internationally, there are mixed views on collaboration between community pharmacies and general practice-based pharmacists. Studies in Canada and Australia have reported good relationships and mutual support [44, 45]. Other Australian studies, in contrast, have described community pharmacists’ reluctance to collaborate or skepticism that pharmacists’ integration might disrupt existing relationships between practices and pharmacies [42, 43]. In England, one study revealed significant tensions between community and general practice-based pharmacists stemming from professional hierarchy and competing, business-related interests [46]. Our findings, however, are predominated by positive experiences and contradict experimentally found or anecdotal frictions. A potential reason for the difference between our findings and those of the other English study is that their participants included general practice-based pharmacists not part of the scheme. Our community pharmacy-based participants had to deal with pharmacists in the scheme who might have been more willing and ready to collaborate with colleagues from community due to their training that includes mandatory sessions on how to build relationships with community pharmacies [52]. Indeed, a national survey of all pharmacists integrated into general practice in the first phase of the scheme highlighted increasing liaison with community pharmacies [9].

### Implications

The study has several implications. There is a need to:

- Appropriately educate community pharmacy teams (for example, via shadowing opportunities) about general practice-based pharmacists’ scope of practice and establish formal, regular meetings between community pharmacy and general practice staff.
- Update community pharmacy teams, in a timely and detailed manner, about any future framing of pharmacists’ presence in general practice (such as expansion of presence or modifications in roles).
- Record the number of interactions with community pharmacies amongst the impact measures for general practice-based pharmacists.
- Establish direct telephone lines or bleepers for pharmacists covering multiple general practices.

### Strengths and limitations

To our knowledge, this is the first study investigating community pharmacy teams’ experiences of general practice-based pharmacists. The qualitative design allowed for an in-depth and thorough understanding of participants’ views. The study captured the experiences of the whole team in community pharmacies as participants included pharmacists, pharmacy technicians and pre-registration pharmacists. Findings account for various levels of working experience in community pharmacy as well as different pharmacy types. Although findings primarily apply to the UK, individual elements will also inform international efforts to integrate pharmacists into general practice such as those in Australia, Canada, New Zealand, Netherlands and the USA.

A limitation of the study is that participants were solely recruited from one area. There might therefore have been additional views of community pharmacy teams in other areas, arising from different general practice-based pharmacists’ employment models and roles. The inclusion, however, of a large number of participants ensured identification of a wide range of experiences. The findings are, therefore, not generalisable but many insights might be transferable to other similar settings. Although the presence of multiple interviewers might translate to some differences in the way interviews were conducted, the use of a common interview schedule provided standardisation in the depth and breadth of topics covered. Researchers followed a reflexive approach throughout data analysis and interpretation by ignoring any personal experiences. Some unavoidable personal assumptions during data categorisation might, however, still exist.

## Conclusions

The current study revealed the potential impact of general practice-based pharmacists on community pharmacies. No significant perceived opposition, from community pharmacy teams, to the scheme was found. Beside benefiting patients and GPs, which was the main driver behind the scheme, pharmacists’ integration into general practice has the potential to streamline the workload of community pharmacy teams and enhance their relationships with practices by enabling communication at a pharmacist-to-pharmacist level. This is an important outcome in light of the recently announced initiatives to better link general practice services with the rest of community healthcare services in the UK. Findings will therefore inform delivery of the NHS Long Term Plan, so that any framing of pharmacists’ presence in general practice includes the needs of community pharmacies. Results will also assist any international policy that is setting out to integrate pharmacists into general practice on how to better integrate care through communication between general practice-based pharmacists and community pharmacies.

## Data Availability

The datasets generated and analysed during the current study are not publicly available because that would compromise participants’ anonymity and the researchers are still publishing findings. Datasets, however, are available from the corresponding author on reasonable request.

## Abbreviations

GP: General Practitioner
GPhC: General Pharmaceutical Council
MUR: Medicine Use Review
NHS: National Health Service
NMS: New Medicine Service
PCN: Primary Care Network
